# Comment on “Independent corroboration of monitor unit calculations performed by a 3D computerized planning system” [J. Appl. Clin. Med. Phys. 1, 120‐125 (2000)]

**DOI:** 10.1120/jacmp.v2i2.2618

**Published:** 2001-03-01

**Authors:** Dimitris N. Mihailidis

**Affiliations:** ^1^ Palmetto‐Richland Memorial Hospital 7 Richland Medical Dr., Suite 104 South Carolina Cancer Center Columbia, SC 29203 dimitris

## Abstract

PACS number(s): 87.53.–j, 87.66.–a

To the Editor,

I read with great interest the article by Leszczynski and Dunscombe published recently [“Independent corroboration of monitor unit calculations (MU) performed by a 3D computerized planning system”; J. Appl. Clin. Med. Phys. 1, 120–125 (2000)]. I strongly agree with the authors that checking the monitor unit calculations produced by a treatment planning system (TPS), especially by a sophisticated 3D one, should be integrated with a comprehensive quality assurance program in routine clinical practice.^1^ However, I feel that the authors' method for performing these checks was unclear and, in some cases, insufficient to accurately verify MUs.

The authors compared TPS MUs and “hand calculation” MUs (MS‐Excel spreadsheet) for specific anatomical treatment sites. The meaning of this comparison is unclear, because they did not explain what kind of clinical setups they use in their center for these sites. Although they recognized that there is a difference in complexity between setups for different sites, the actual treatment technique should be described in order to help the reader make a comparison with his/her own techniques. As an example, there is a different degree of dosimetric complexity when asymmetric jaws in length and width are used to treat the tangential breast rather than simple symmetric fields. Although both techniques may require wedges, in the former case the prescription point will be at an off‐axis location (relative to the blocked isocenter) in both wedged and nonwedged directions.

The authors have also not included several specific details of the methodology by which the treatment planning system calculates MUs. For example, experience from other 3D (Ref. 2) TPS's has shown that point doses and eventually MU calculations can be altered by the calculation grid size, the beam modeling parameters, and by how well data, such as large wedged profiles, are calculated and fitted by the planning algorithm during commissioning.^3^ These calculations are made even more complex with the inclusion of inhomogeneity corrections. It is unclear in their article what is required of these parameters to obtain this accuracy and how variations will effect the results of Table I.^4^

In some circumstances the authors' formalism may not be sufficiently accurate “to perform its intended function.” For example, the authors chose not to use off‐axis correction factors for open fields, even though they use these corrections for wedged fields. Although off‐axis corrections are very large for wedged fields, they can be quite substantial for open fields as well. Even in the case of the supraclavicular treatment field referenced in their article, off‐axis corrections are between 1.025‐1.040 for a 6‐MV beam (Varian 2100C/D) at 3‐cm depth and 3‐8‐cm off‐axis distances. Of course, these corrections will be even larger for greater off‐axis distances.^5^ Additionally, the authors decision not to include hardening corrections for wedged fields will also compromise accuracy. Those corrections can be as high as 1.020 for an 18‐MV beam (Varian 2100C/D) and 15‐cm depth. Some information about which therapy equipment is used at their center would help interested readers estimate the magnitude of those correction factors.

In order to implement an independent and consistent method for validating the MU calculations produced by a sophisticated 3D treatment planning system, such as the HELAX TMS, one should make sure to include all significant parameters involved in the independent MU calculation formalism. In the independent MU calculation that the authors presented [Eq. (1)], several approximations were made that eventually may have compromised the accuracy of their calculations. Following the same formalism^6^ with the authors, one should have:

(a) Explicitly separated the collimator scatter $\left(S_{c}\right)$ from the phantom scatter factor $\left(S_{p}\right)$ (it is unclear whether this was done by the authors, and I am sure that complex beam shaping is used routinely in 3D dose calculations);
(b) included depth and field size dependent wedge factors;^7^
(c) included a depth and distance dependent off‐axis ratio (OAR) correction factor^4^ for the unwedged fields; and
(d) included an ${\text{OAR}}_{w}$ (wedged‐OAR) if a wedged field is used (for the off‐axis prescription points). Actually, even the use of wedged beam profile values in place of an ${\text{OAR}}_{w}$ were shown to be inadequate especially for larger wedges and large off‐axis distances.^8^

Following these steps one can perform manual MU calculations and verify the results of a treatment planning system in a consistent way.
